# Film-forming, stable, conductive composites of polyhistidine/graphene oxide for electrochemical quantification of trace Pb^2+^

**DOI:** 10.1039/d3ra00848g

**Published:** 2023-05-19

**Authors:** Zhe-Han Yang, Xin Lei, Guangming Jiang, Xianming Zhang

**Affiliations:** a Engineering Research Center for Waste Oil Recovery, Technology and Equipment of Ministry of Education, Chongqing Key Laboratory of Catalysis and Functional Organic Molecules, College of Environment and Resources, Chongqing Technology and Business University Chongqing 400067 China yzhzn89@ctbu.edu.cn +86-023-62768056

## Abstract

Nanomaterials with unique properties, such as good film-formation and plentiful active atoms, play a vital role in the construction of electrochemical sensors. In this work, an *in situ* electrochemical synthesis of conductive polyhistidine (PHIS)/graphene oxide (GO) composite film (PHIS/GO) was designed to construct an electrochemical sensor for the sensitive detection of Pb^2+^. Herein, GO as an active material can directly form homogeneous and stable thin films on the electrode surface because of its excellent film-forming property. Then GO film was further functionalized by *in situ* electrochemical polymerization of histidine to obtain plentiful active atoms (N). Due to strong van der Waals forces between GO and PHIS, PHIS/GO film exhibited high stability. Furthermore, the electrical conductivity of PHIS/GO films was greatly improved by *in situ* electrochemical reduction technology and the plentiful active atoms (N) in PHIS are profitable for adsorbing Pb^2+^ from solution, tremendously enhancing the assay sensitivity. With the above unique property, the proposed electrochemical sensor showed high stability, a low detection limit (0.045 μg L^−1^) and a wide linear range (0.1–300 μg L^−1^) for the quantification of Pb^2+^. The method can also be extended to the synthesis of other film-forming nanomaterials to functionalize themselves and widen their potential applications, avoiding the addition of non-conductive film-forming substances.

## Abstract 2

*In situ* electrochemical synthesis of poly-histidine on graphene oxide film-modified electrode (PHIS/GO/GCE) was conducted to construct an electrochemical chemical sensor for Pb^2+^ assay. This method could be extended to the synthesis of other film-forming nanomaterials.

## Graphical abstract

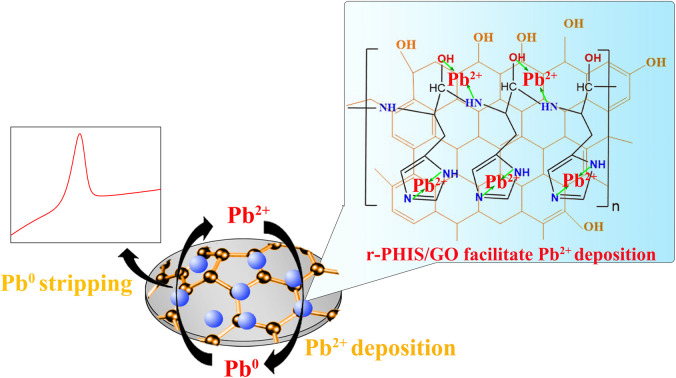

## Introduction

The United States Environmental Protection Agency (U.S. EPA) reported that 10–20% of adults and 40–60% of infants are exposed to Pb *via* drinking water and food.^1,2^ Even at trace concentrations, heavy metals pose a major threat to human health because of their inherent properties, including bioaccumulation and non-biodegradability as well as toxicity.^3,4^ The World Health Organization (WHO) has established a guideline that limits the total Pb concentration in drinking water to 10 ppb.^5^ Therefore, it is essential to accurately assay the total amount of Pb in drinking water and food. Commonly used assay methods include flame atomic absorption spectrometry,^6^ inductively coupled plasma (ICP)-atomic emission spectrometry,^7^ ICP-mass spectrometry,^8^ and potentiometric ion selective electrodes.^9^ Among these, the electrochemical method has aroused a lot of attention due to various advantages, such as simplicity, high sensitivity, low cost and the choice of functional materials.^10,11^ Because the stable attachment of functional materials onto the electrode plays a key role in guaranteeing the excellent performance of an electrochemical assay for Pb^2+^, the functional materials need to mix with film-forming substances such as Nafion to enhance stability.^12,13^ However, film-forming substances are organic polymers which dramatically hinder electron transport, decreasing the performance of the electrochemical assay for Pb^2+^.^14^ Therefore, a method that can attach functional materials onto an electrode surface free of film-forming substance is urgently being sought.

Graphene oxide (GO) as a dimensional layered material has been widely applied in various fields, such as electronics, energy, composite materials and bio-applications for its excellent electrical, thermal, and mechanical properties.^15–18^ Moreover, unlike other large sp^2^-conjugated structures, GO can easily form a homogeneous dispersion because it has rich –OH and –COOH groups on the GO surface.^19^ More importantly, GO can be solution-processed into homogeneous and stable thin films, which endow it with the ability to modify electrode materials free of film-forming substances.^15,20,21^ However, oxygen-containing functional groups (especially C=O, –COOH) have a strong electron absorption ability, leading to negative effects on electrical conductivity, decreasing the performance of the modified electrode.^22^ Therefore, many attempts have been made to enhance the electrical conductivity and improve the performance of GO-modified electrodes. For example, l-cysteine that contains –SH and –NH_2_ was chosen as a functional molecule to modify GO by forming an amide bond between –NH_2_ in l-cysteine and –COOH in GO for the detection of Pb^2+^.^23^ The polypyrrole was functionalized on the GO surface to introduce N atoms for adsorbing target metal ions.^24^ Although the performance of the above-modified electrode was improved to some extent compared with the GO-modified electrode, its conductivity was not obviously enhanced due to the still existing amount of electron-withdrawing group (C=O).^25^ Therefore, a method that can enhance electrical conductivity as well as improve the performance of the GO-modified electrode is still needed.

**Scheme 1:**
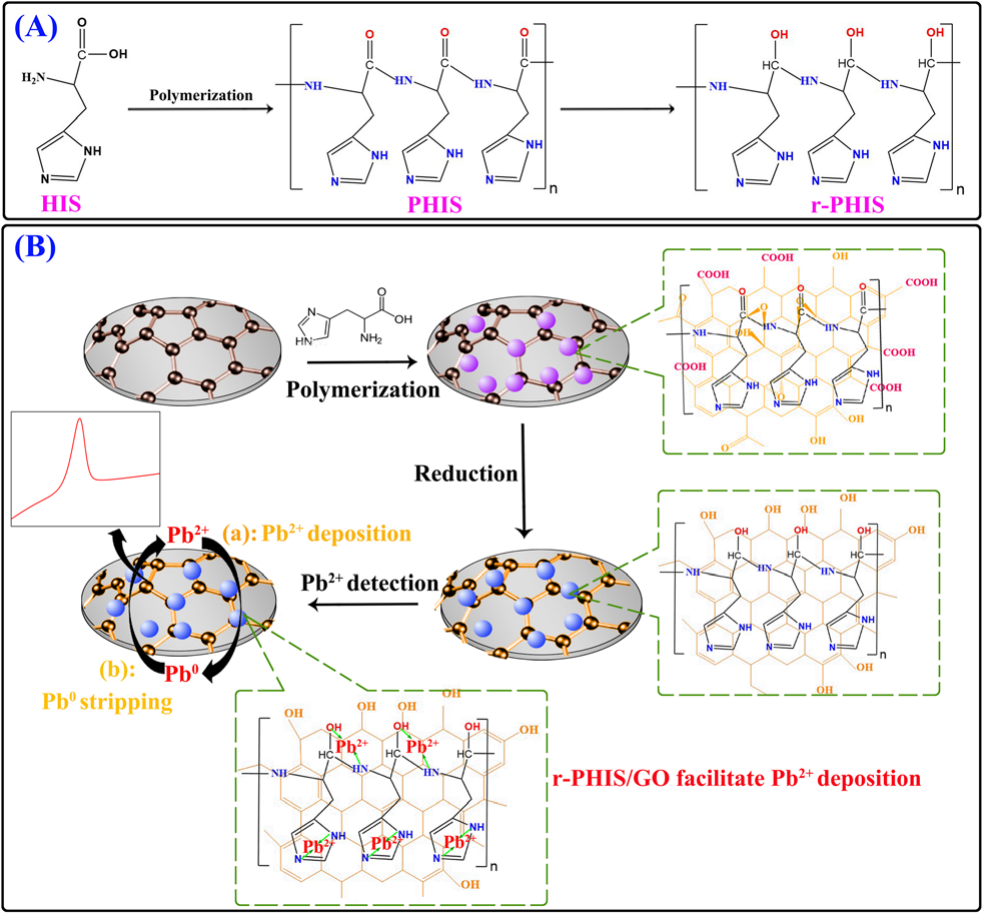
(A) Schematic representation of molecular structure before and after polymerization and electrochemical reduction. (B) Schematic illustration of the modification process of an electrochemical sensor and its application in the detection of Pb^2+^.

In this work, an *in situ* electrochemical synthesis of polyhistidine (PHIS)/GO film coupled with an electrochemical reduction strategy was designed to construct an electrochemical sensor for a Pb^2+^ assay. Firstly, a GO dispersion solution was dropped onto the electrode surface and dried at room temperatures to obtain a GO film-modified electrode (GO/GCE). Then, *in situ* electrochemical polymerization was conducted to obtain PHIS-functionalized GO/GCE (PHIS/GO/GCE).^26^ Next, the –C=O group in the PHIS/GO film was removed under the action of constant potential electrolyzation, achieving the reduction of PHIS/GO (r-PHIS/GO). Three outstanding properties of r-PHIS/GO endow the proposed electrochemical chemical sensor with excellent performance for the monitoring of Pb^2+^: (1) With its film-forming property, GO can directly form homogeneous and stable thin films at room temperature, avoiding the addition of a non-conductive film-forming substance. Moreover, due to the large sp^2^-conjugated structures, GO can help stabilize the PHIS film on the electrode for strong van der Waals forces between GO and PHIS. (2) Because of successive electrochemical reduction, the removal of the –C=O group in PHIS/GO film would increase the electrical conductivity. (3) PHIS is a polyaminoacid-bearing imidazole group (p*K*_a_ = 6.0) that can provide plentiful active atoms (N) to adsorb Pb^2+^, enhancing the sensitivity of the assay. As a result, the proposed electrochemical sensor showed high stability, a low detection limit (0.045 μg L^−1^) and a wide linear range (0.1–300 μg L^−1^) for the quantification of Pb^2+^ (Scheme 1).

## Experimental section

### Reagents and instruments

Graphite oxide (GO) was obtained from Nanjing Xianfeng Nano Co. (Nanjing, China). Pb(NO_3_)_2_, Na_2_HPO_4_, NaH_2_PO_4_, KCl, sodium acetate (NaAc) and acetic acid (HAc) were purchased from Chengdu KeLong Chemical Reagents (Chengdu, China). HIS was obtained from Aladdin (Shanghai, China).

Electrochemical measurements were carried out on a CHI 660E electrochemical workstation (Shanghai Chenhua Instrument Co., Ltd, China) using a three-electrode system: a platinum wire auxiliary electrode, a saturated calomel reference electrode and a glassy carbon electrode (GCE, *Φ* = 3 mm) as the working electrode. X-ray photoelectron spectroscopy (XPS) data was obtained on the surface of the samples using an electron spectrometer (Thermo Escalab 250Xi, Thermo Scientific, USA) and fitted with XPS PEAK software. The morphology of the nanomaterials was characterized by a high-resolution scanning electron microscope (SEM, JSM-7001F INCA X-MAX, Japan). Cyclic voltammetry (CV) was performed in 5.0 mM [Fe(CN)_6_]^3−/4−^ with a scanning potential from −0.2 to 0.6 V at a scan rate of 50 mV s^−1^. Electrochemical impedance spectroscopy (EIS) was performed at 5.0 mM [Fe(CN)_6_]^3−/4−^ with parameters including a 50 mV s^−1^ sweep rate, 0.22 V initial electric potential, 0.1 Hz low frequency, 100 000 Hz high frequency and a 2 s pulse period.

### Preparation of r-PHIS/GO modified electrode

Firstly, GCEs were pre-treated according to the reported protocol.^22^ 10 mg of GO was dispersed into 10 mL of double-distilled water and ultrasonicated to form a homogeneous dispersion solution. After that, 5 μL of the prepared GO solution was dropped onto the cleaned GCE and dried at room temperature to obtain the GO layer (GO/GCE). The GO/GCE was placed in a 0.1 M phosphoric acid buffer solution (PBS, pH 9.0) containing 0.02 M histidine (monomer) and CV was conducted with six cycles between −0.8 and +2.0 V (*vs.* Hg/Hg_2_Cl_2_) at a scan rate of 100 mV s^−1^, obtaining polyhistidine modified GO/GCE (PHIS/GO/GCE). Following that, the PHIS/GO/GCE was immersed in 0.5 M KCl solution, and successive electrochemical reduction was performed by chronoamperometry at a constant potential of −1.3 V for 1 h to achieve a reduced PHIS/GO layer (r-PHIS/GO).

### Pb^2+^ quantification

Square wave anodic stripping voltammetry (SWASV) was used to analyze the concentrations of Pb^2+^ in HAc buffer (pH 5.0) with various concentrations of Pb^2+^, where Pb^2+^ was electrodeposited at 0.8 V for 240 s, and then stripped by SWV from −1.1 to −0.1 V with a step size of 5 mV, pulse width of 0.2 s and amplitude of 50 mV. At the end of each detection test, a 1 V potential was applied on the working electrode for 100 s in order to remove the deposited residual species from its surface.

## Results and discussion

### Characterization of the as-prepared r-PHIS/GO

**Fig. 1:**
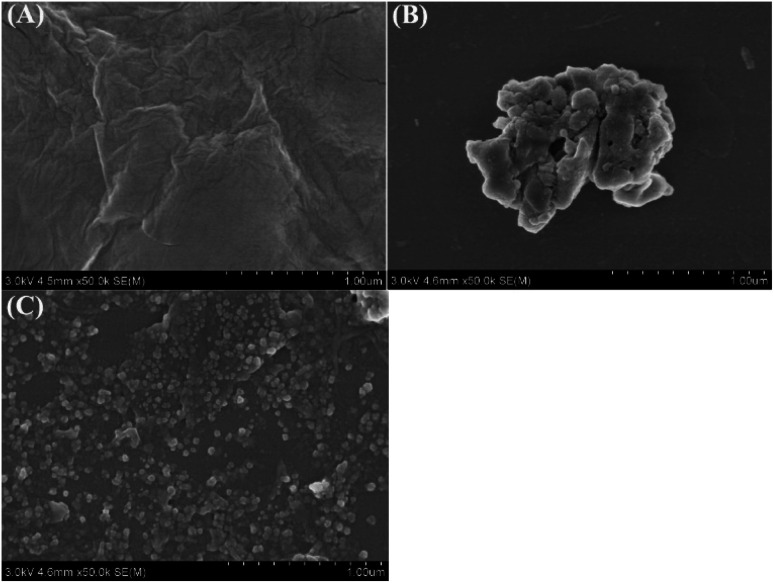
SEM images of GO (A), PHIS (B) and r-PHIS/GO (C).

The morphologies of GO and r-PHIS/GO were characterized using SEM. As can be seen in Fig. 1A, GO showed restacked layers and wrinkles in some regions, which may be ascribed to electronic repulsion among the soft layers. Fig. 1B shows the morphologies of PHIS prepared by direct deposition onto the GCE surface. It can be seen that the PHIS is composed of large irregular lumps. When PHIS was deposited on the GO surface, PHIS showed smaller nano-scale irregular particles, dispersed uniformly on the GO surface, indicating that GO helps to form uniformly distributed PHIS.

### Elemental analysis of r-PHIS/GO

**Fig. 2:**
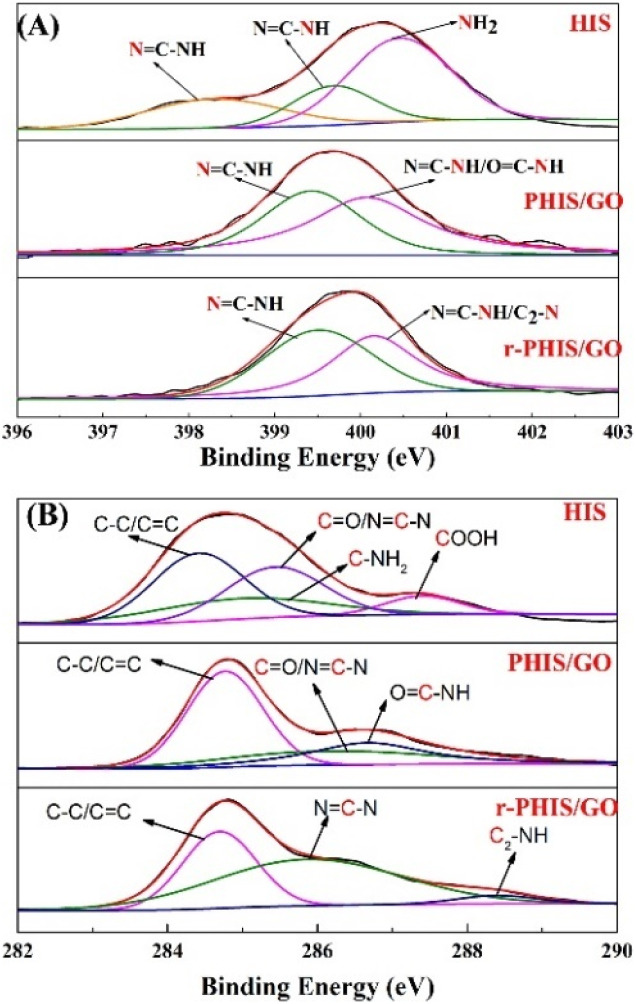
(A) N 1s XPS spectra of pure HIS, PHIS/GO, r-PHIS/GO; (B) C 1s XPS spectra of pure HIS, PHIS/GO, r-PHIS/GO.

Photo electron spectra were performed to investigate the change in the elements during the preparation process of r-PHIS/GO. The detailed N 1s peaks of pure HIS, PHIS/GO and r-PHIS/GO are shown in Fig. 2A. The spectrum of pure HIS can be split into three peaks at around 398.3, 399.7 and 400.5 eV. The peaks at 398.3 eV and 399.7 eV were attributed to the imidazole group (N=C–N and N=C–N). The peak at 400.5 eV was assigned to amino functions. When the electrochemical polymerization of HIS onto the GO/GCE surface was conducted, only two peaks at 399.1 eV and 400.1 eV could be observed. This result was due to the fact that the reaction between amino functions and carboxyl was driven at −1.3 V with the formation of O=C–NH, and electrochemical polymerization caused the imidazole group peaks (N=C–N and N=C–N) to shift to higher binding energy. After electrochemical reduction, no obvious change in imidazole group peaks was observed. Similarly, for a better comparison, XPS analysis of C 1s was further investigated (Fig. 2B). The four XPS peaks of C 1s for pure HIS could be observed at 284.4 eV, 284.9 eV, 285.4 eV and 287.4 eV, which were attributed to C–C/C=C, C=O/N–C=N, C–NH_2_ and COOH, respectively. However, the peaks corresponding to COOH disappeared after electrochemical polymerization, suggesting that COOH was transferred to other groups (O=C–NH). Furthermore, a new peak at 288.3 eV could be observed after electrochemical reduction, which implies the formation of H–N–C_2_ bonds.

### Characterization of the stepwise fabrication of the chemical sensor

**Fig. 3:**
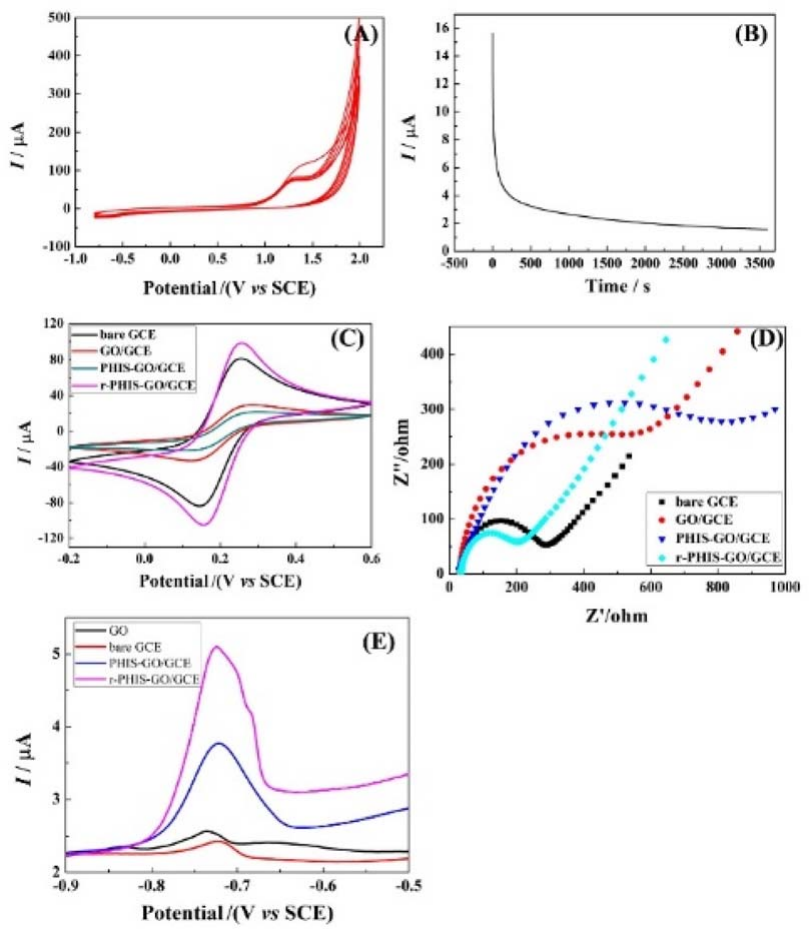
(A) The cyclic voltammograms of GO/GCE in 0.1 M PBS (pH 9.0) containing 0.02 M HIS (monomer). (B) *i*–*t* curve of PHIS/GO/GCE under 1.3 V for 1 h in 0.5 M KCl. Electrochemical characterizations of different modified electrodes: (C) CV and (D) EIS in the [Fe(CN)_6_]^3−/4−^ system. (E) SWASV curves of bare GCE, GO/GCE, PHIS-GO/GCE and r-PHIS-GO/GCE in 0.1 M acetate buffer solution (pH 5.0) containing 100 μg L^−1^ Pb^2+^.

Fig. 3A shows the cyclic voltammograms recorded in 0.1 mol L^−1^ PBS (pH 9.0) containing 0.02 mol L^−1^HIS (monomer) using GO/GCE. An anodic peak at 1.25 V could be observed due to oxidation and deposition of the HIS monomers, indicating that HIS could be successfully polymerized. Fig. 3B shows the *i*–*t* curve of PHIS/GO/GCE under 1.3 V for 1 h in 0.5 M KCl. When *t* > 0, a large current can be observed, and then the current decreases rapidly. With an increase in electrolysis time, the current gradually decreases and finally remains relatively stable, indicating that the electrochemical properties of the electrode materials tend to be stable. To characterize the stepwise fabrication of r-PHIS/GO/GCE, electrochemical measurements (CV and EIS) were conducted. As shown in Fig. 3C, an obvious decrease in CV redox peaks in GO/GCE could be observed in comparison with GCE, indicating that GO was successfully attached onto the GCE surface because amounts of O-containing groups on GO surface hindered electron transfer. After *in situ* polycondensation in HIS solution, a further decrease in peak current was obtained, indicating that PHIS was successfully deposited onto the GO/GCE surface. To improve the conductivity of PHIS/GO/GCE, constant voltage scanning was performed and the peak currents of r-PHIS/GO/GCE were apparently increased, which indicated that g-constant voltage scanning was beneficial for improving the conductive property of PHIS/GO/GCE. In addition, EIS was conducted to further verify the CV results. As shown in Fig. 3D, GO/GCE exhibited bigger impedance than GCE. An increase in impedance was obtained after HIS polymerization. A dramatic decrease in EIS value was achieved after electrochemical reduction. The EIS characterization result is consistent with the CV results, suggesting the successful preparation of r-PHIS/GO/GCE.

### Electrochemical response of different modified electrodes

To assess the superiority of r-PHIS/GO/GCE for Pb^2+^ assay, four modified electrodes, bare GCE, GO/GCE, PHIS/GO/GCE, and r-PHIS/GO/GCE, were prepared for a comparison of the response to the same concentration of Pb^2+^. Fig. 3E shows the stripping current curves of the above four electrodes in 0.1 M HAc buffer solution (pH 5.0) containing 100 μg L^−1^ Pb^2+^. The red line and black line show the stripping peak current at bare GCE and GO/GCE, respectively. It can be seen that both electrodes show weak current responses. Moreover, a remarkable increase in the stripping peak current for Pb^2+^ ions was obtained at PHIS/GO/GCE (blue line), indicating that PHIS was beneficial for absorbing more metal ions. Furthermore, compared with PHIS/GO/GCE, the current response of r-PHIS/GO/GCE was obviously increased (red line), which may be ascribed to the excellent electrical conductivity of r-PHIS/GO/GCE. Therefore, r-PHIS/GO/GCE is superior for a Pb^2+^ assay.

### Optimization of conditions

**Fig. 4:**
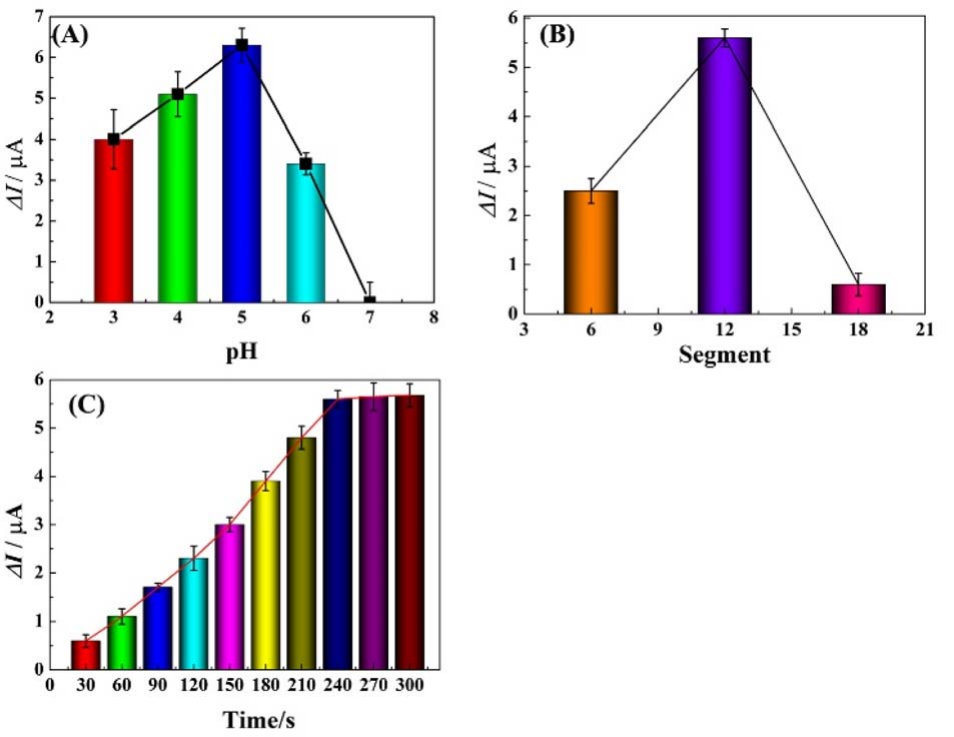
Influence of pH value (A), deposition potential (B) and accumulation time (C) on the stripping peak currents.

To achieve optimal performance for Pb^2+^ detection, the assay conditions, including the pH of the detection solution, polymerization time of HIS and deposition time of Pb^2+^ were optimized. The stripping responses of Pb^2+^ in HAc buffer solution of various pH (ranging from 3.0 to 7.0) were investigated using SWASV. As shown in Fig. 4A, the peak current response was enhanced from pH 3.0 to 5.0 and decreased from 6.0 to 7.0, which was ascribed to the competitive binding between proton ions and metal ions to the donating atoms in lower pH solution and the hydrolysis of Pb^2+^ in high pH solution. Therefore, pH 5.0 was used as the optimized value and applied in the following studies.

Electrochemical characterization verifies that PHIS is non-conductive; thus the amount of PHIS should be optimized by controlling the polymerization time (CV measurement used segments to mean polymerization time) and comparing the stripping current responses. As shown in Fig. 4B, the effect of polymerization time in the range of 6 to 18 segments was investigated. It can be seen that the current response of 100 μg L^−1^ Pb^2+^ was enhanced from 6 segments to 12 segments and then decreased from 12 segments to 18 segments. Thus, the optimal polymerization time was 12 segments.

The stripping current response was influenced by the deposition time of Pb^2+^; thus the effect of deposition time on the stripping current response at r-PHIS/GO/GCE was further investigated using SWASV. From Fig. 4C, we can see that the current response of 100 μg L^−1^ Pb^2+^ was enhanced with increasing deposition time from 30 s to 240 s and then tended to plateau from 240 s to 300 s. Thus, we chose 240 s as the optimal deposition time.

### Control of electron transfer process

**Fig. 5:**
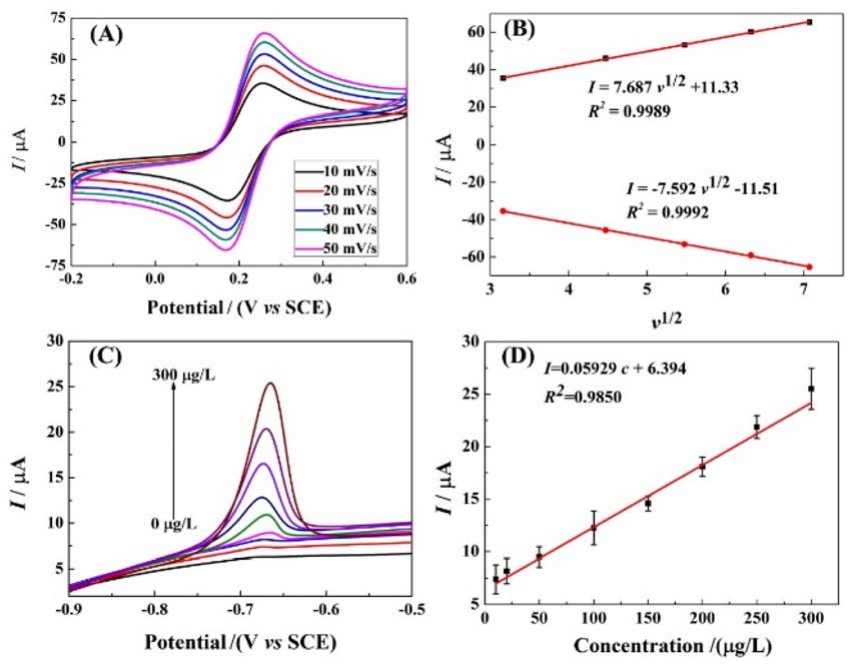
(A) CV response in PBS buffer containing 5.0 mM [Fe(CN)_6_]^3−/4−^ at different scan rates: 10, 20, 30, 40, and 50 mV s^−1^; (B) calibration of linear relationship between the CV currents and scan rates. (C) SWASV response of various Pb^2+^ concentrations at g-C_3_N_4_/r-GO/GCE in HAC buffer (pH 5.0); (D) calibration of linear relationship between the SWASV currents and the concentrations of Pb^2+^.

In order to further investigate the electron transfer process on r-PHIS/GO/GCE, the CV current at different scan rates was investigated. As shown in Fig. 5A, the redox peak currents increased with an increment in scan rates, accompanied by an increase in potential gap. Moreover, the oxidation and reduction peak currents were linearly proportional (*R*^2^ = 0.9989, 0.9992) to the square root of the scan rate (*v*^1/2^) in the range from 10 to 50 mV s^−1^. The linear equations are *I*_pa_ (μA) = 8.678*v*^1/2^ + 11.33 and *I*_pc_ (μA) = −7.592v^1/2^ − 11.51 (Fig. 5B). The above results suggest that the electron transfer process on r-PHIS/GO/GCE was diffusion-controlled. Therefore, the stripping peak current value is relevant to the concentration of Pb^2+^.

### Performance of r-PHIS/GO/GCE for Pb^2+^ detection

Under the optimal conditions, the performance of r-PHIS/GO/GCE for Pb^2+^ detection was assessed in HAc buffer (pH 5) containing various concentrations of Pb^2+^ by SWASV. As shown in Fig. 5C, increased oxide currents were observed with the increase in Pb^2+^ concentration and were shown to be linear with concentration in the range of 1.0–300 μg L^−1^. The equation of the calibration curve was *I* (μA) = 0.05929c (μg L^−1^) + 6.394 with correlation coefficient (*R*^2^) 0.9850 (Fig. 5D). The limit (3S/N) was 0.045 μg L^−1^. Furthermore, Table 1 shows a comparison of the performance of r-PHIS/GO/GCE for Pb^2+^ with some previous reported work. The results suggested that the performance of r-PHIS/GO/GCE for Pb^2+^ was acceptable and competitive.

**Table 1:** Comparison of r-PHIS/GO/GCE for Pb^2+^ assay with previous reported work

Electrode materials	Method	Detection limit (μg L^−1^)	Detection range (μg L^−1^)	Ref.
BiMOF/CP	SWASV	2.7	8.5–34	27
Au@Nb_4_C_3_T_*x*_	DPASV	0.83	2.1–1035	28
Au@SiO_2_@Fe_3_O_4_/NG	DPASV	0.60	5–80	29
ZnO-RGO	DPASV	0.090	0.5–10	30
g-C_3_N_4_/r-GO	SWASV	0.15	1–300	25
NH_2_-MIL-53(Cr)	SWASV	6.0 × 10^−3^	8.3 × 10^−2^ to 1.6 × 10^3^	31
2D MoS_2_ nanofilm	SWASV	0.30	0–20	1
UiO-66-NH_2_	DPASV	0.030	1.0 × 10^−3^ to 1.0 × 10^3^	32
Boron nitride	LSASV	0.15	4.0–1.5 × 10^3^	33
r-PHIS/GO	SWASV	0.045	0.1–300	This work

### Specificity and stability

**Fig. 6:**
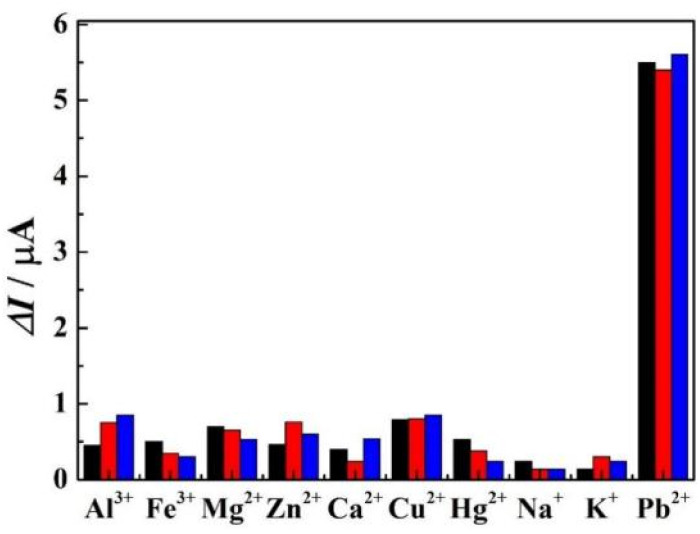
The specificity of r-PHIS/GO/GCE for Pb^2+^ detection.

The specificity of r-PHIS/GO/GCE was assessed by challenging it against other usual metal ions, including, Al^3+^, Fe^3+^, Cu^2+^, Mg^2+^, Zn^2+^, Ca^2+^, Hg^2+^, K^+^, and Na^+^. The concentration of the interfering ions was 5 mg L^−1^ and the concentration of target Pb^2+^ was 50 μg L^−1^. As shown in Fig. 6, the results demonstrated that a 100-fold high concentration of interfering metal ions showed minimal current response, but a large current response was exhibited at 50 μg L^−1^ Pb^2+^ (peak current change <7%), suggesting r-PHIS/GO/GCE has excellent selectivity for a Pb^2+^ assay. Moreover, the stability was assessed by storing r-PHIS/GO/GCE with physical protection and measuring the same concentration of Pb^2+^ every four days. After storage for 20 days, the current response was 89.1% of the initial response, indicating that r-PHIS/GO/GCE showed relatively excellent stability for a Pb^2+^ assay.

### Analysis of real samples

The prepared r-PHIS/GO/GCE was further challenged to detect Pb^2+^ from industrial wastewater. Prior to detection, all of the samples were filtered through a 0.45 mm membrane. The four industrial wastewater samples (pH 5.0) were detected by the r-PHIS/GO/GCE and ICP methods, respectively. The percentage variance between the measured results of the above two methods for the four samples was within 9.5%, revealing that the prepared r-PHIS/GO/GCE was suitable for the practical detection of real samples with acceptable accuracy (Table 2).

**Table 2:** Analysis of real samples using the electrochemical method and ICP

No.	Electrochemical method	ICP	Percentage variance (%)
1	2.57	2.7	9.5
2	10.5	11	4.5
3	89.6	83.9	6.7
4	112.3	122.9	8.6

## Conclusions

In conclusion, the *in situ* electrochemical synthesis of r-PHIS/GO/GCE was designed to construct a chemical sensor for a Pb^2+^ assay. With the film-forming property of GO and *in situ* polymerization of PHIS, r-PHIS/GO/GCE avoids using an extra film-forming substance to stabilize nanomaterials on the electrode surface. GO also helps stabilize PHIS film on an electrode for strong van der Waals forces between GO and PHIS. Because of the removal of the –C=O group in PHIS/GO film by electrochemical reduction and the plentiful active atoms (N) in PHIS, r-PHIS/GO/GCE ensures high sensitivity for Pb^2+^ detection. Furthermore, the method can also be extended to the synthesis of other film-forming nanomaterials to functionalize themselves and widen their potential applications.

## Conflicts of interest

The authors declare no competing financial interest.

## Supplementary Material
